# Short-tandem repeat analysis in seven Chinese regional populations

**DOI:** 10.1590/s1415-47572010000400002

**Published:** 2010-12-01

**Authors:** Xing-bo Song, Yi Zhou, Bin-wu Ying, Lan-lan Wang, Yi-song Li, Jian-feng Liu, Xiao-gang Bai, Lei Zhang, Xiao-jun Lu, Jun Wang, Yuan-xin Ye

**Affiliations:** 1 Department of Laboratory Medicine, West China Hospital, Sichuan University, Chengdu, SichuanP.R. China; 2 The Police Station of Wenzhou, Wenzhou, ZhejiangP.R. China; 3 The Police Station of Chengdu, Chengdu, SichuanP.R. China

**Keywords:** forensic medicine, population genetics, short-tandem repeat, human evolutionary history, genetic distance

## Abstract

In the present study, we investigated the application of 13 short tandem repeat (STR) loci (D13S317, D7S820, TH01, D16S539, CSFIPO, VWA, D8S1179, TPOX, FGA, D3S1358, D21S11, D18S51 and D5S818) routinely used in forensic analysis, for delineating population relationships among seven human populations representing the two major geographic groups, namely the southern and northern Chinese. The resulting single topology revealed pronounced geographic and population partitioning, consistent with the differences in geographic location, languages and eating habits. These findings suggest that forensic STR loci might be particularly powerful tools in providing the necessary fine resolution for reconstructing recent human evolutionary history.

## Introduction

The present Chinese population of around 1.4 billion is primarily divided by the Yellow River into two large groups, the southern and the northern, with diverse languages and eating habits. There is thus an immense scope to study the processes of anthropological subdivisions and microevolutionary effects in different populations groups of China. However, the traditional structure of Chinese populations is facing the imminent threat of disintegration through urbanization and increasing communication, with the consequential gene flow between subcastes through marriages. Therefore, there is a need for understanding local traditional population structure and its role in shaping human genome diversity.

A large-scale survey of autosomal variation in an ample geographic sample of human Asian populations has shown that, apart from geography, genetic ancestry is strongly correlated with linguistic affiliations (The HUGO Pan-Asian SNP Consortium 2009). A distinction between northern and southern Chinese populations (Han and minority alike) has been observed on analyzing genetic markers (Zhao and Lee, 1989; Chu *et al.*, 1998). Short tandem repeat (STR) loci are highly polymorphic loci in the human genome, are relatively small in size, and can be analyzed in a multiplex PCR fashion. Many population genetic studies have investigated the polymorphism profile of the STR system in Chinese Han populations, this including the loci D13S317, D7S820, TH01, D16S539, CSFIPO, VWA, D8S1179, TPOX, FGA, D3S1358, D21S11, D18S51 and D5S818 (Cai *et al.*, 2005; Deng *et al.*, 2007). In the present study, these 13 STR loci in seven Chinese regional populations, comprising 3 northern, (Henan, Beijing and Tianjin) and 4 southern (Sichuan, Fujian Guangdong, and Zhejiang), were analyzed by way of capillary electrophoresis on 3100 genetic analyzers.

Based on the population data of these STR polymorphisms, the forensic parameters of the respective loci were calculated in order to estimate their value in genetic identity testing. Furthermore, genomic affinities among the diverse regional population groups were evaluated. The current study contributed to supplementing the ever-increasing population-information database worldwide.

## Materials and Methods

###  Sample preparation

Whole blood was obtained by venipuncture in EDTA-coated vaccutainers from unrelated, consenting donors. Community history and family disease backgrounds were recorded on blood donor cards.

**Figure 1:**
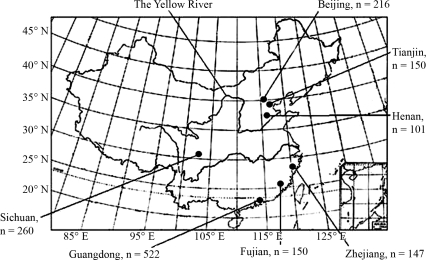
Geographical location of the seven populations in China.

Seven geographically targeted populations, encompassing the major biogeographical zones and representing the two main Han populations (southern and northern), were selected. These included 4 southern, the Sichuan (n = 260, Ying *et al.*, 2005), Fujian (n = 150), Guangdong (n = 522) and Zhejiang (n = 147), and 3 northern, the Henan (n = 101), Tianjin (n = 150) and Beijing (n = 216). Their respective location is shown in Figure 1

DNA was extracted using the Chelex method (Walsh *et al.*, 1991).

###  PCR amplification

PCR amplification was carried out on a thermal cycler, using primers with the same sequences as those in the “PowerPlex 16 System” kit (Krenke *et al.*, 2002). Each PCR reaction was performed with 2.5 μL of template DNA (5-250 ng), 0.5 μM of each primer, 2.5 μL ofTaq buffer (10PCR Buffer, Applied Biosystems), 2 μL of MgCL_2_ (25 μM, Applied Biosystems), 0.5 μL of a dNTPs mix (10 μM PCR nucleotide Mix, Promega), and 1U Taq polymerase (DyNAzyme, DNA Polymerase, Finnzymes) in a total volume of 25 μL. A total of 30 cycles were run, with an initial incubation (preliminary denaturation) step at 96° C for 2 min, followed by 10 cycles of 94 °C for 1 min, 60 °C for 1 min and 70 °C for 1.5 min, followed by 20 cycles of 90 °C for 1 min, 60 °C for 1 min and 70 °C for 1.5 min, ending with a final extension at 60 °C for 30 min.

###  Electrophoresis and analysis

The PCR product (1.5 μL), as well as GeneScan-400HD-ROX Size Standard (Applied Biosystems) (0.5 μL), were added to 24.5 μL of deionized formamide, and subsequently denatured for 3 min at 95 °C. Alleles were then separated by capillary electrophoresis in POP-4 polymer (Applied Biosystems) with the GS STR POP4 D Module (1 mL), using an ABI PRISM 3100 Genetic Analyzer (Applied Biosystems). Samples were injected into the capillaries in batches of 16 samples, directly from the microtitre plate, for 10 s at 3 kV. Electrophoresis was performed at 15 kV and 60 °C for 45 min under routine running conditions. Alleles were identified by means of GeneScan Analysis 3.7 Software (Applied Biosystems), whereupon the analyzed data were automatically genotyped using Genotyper 3.6 Software (Applied Biosystems) and a template specially made for this specific multiplex system. The Peak Amplitude Threshold adopted was more than 150 RFU (relative fluorescence units).

###  Statistical analysis

Individual locus frequency was calculated from the number of each genotype in the sample set. Unbiased estimates of expected heterozygosity were computed as described by Edwards *et al.* (1992). Possible divergence from Hardy-Weinberg equilibrium (HWE) was determined by calculating an unbiased estimate of expected homozygote/heterozygote frequencies (Nei and Roychoudhury, 1974; Chakraborty *et al.*, 1988; ), through likelihood-ratio testing (Weir, 1992; Buscemi *et al.*, 1995). The Chi-square test was applied for comparing the genotype and allelic frequency of each STR locus among the studied populations. We also calculated certain parameters of genetic and forensic interest, *i.e.*, the power of discrimination (Grunbaum *et al.*, 1978), the chance of exclusion (Ohno *et al.*, 1982), polymorphism information content (PIC) (Botstein *et al.*, 1980) and heterozygosity. Distance was estimated using the Nei formula (Nei and Roychoudhury, 1972; Li and Nei, 1977), whereas phylogeny was inferred by UPGMA and Neighbor-Joining methods in Mega 2.1.

## Results

###  Polymorphisms of 13 STR loci in seven Chinese Han populations

Details on polymorphism exhibited at the 13 loci with respect to the allele frequencies in the seven Chinese populations are listed in Tables S1-S13.

Despite the wide range of allelic variation in the 13 STR loci, a discernable pattern depicting mutual geographical affiliation is apparent. Generally speaking, frequency was high in only few alleles (*e.g.*, allele 9 of TH01, allele 14 of VWA, allele 14 of D16S539, allele 30 of D21S11, and allele 10 of TPOX) (Tables S1-S13). 13 STR loci among seven Chinese populations showed similar trends Furthermore, both genotype and allele distribution were not significantly different among the seven Chinese populations (p > 0.05). These results are thought to reflect the influence of gene flow due to geographic proximity.

###  Phenotype distribution and value in forensic application

The distribution of observed allele frequencies in the 13 loci (D13S317, D7S820, TH01, D16S539, CSFIPO, VWA, D8S1179, TPOX, FGA, D3S1358, D21S11, D18S51, D5S818), as well as the results from the various analytical procedures for testing the correspondence of genotype frequencies with Hardy-Weinberg equilibrium, are shown in Tables S1-S13.

All the 13 loci complied with Hardy-Weinberg equilibrium, with no evidence of association of alleles among the 13 loci. The parameters for both forensic efficiency and genetic variability, such as MP, PD, PIC, PE and heterozygosity, were calculated and subsequently listed for each population in the supplementary tables.

###  Analysis of genetic distances

In order to ascertain relationships among the seven Chinese populations, we have calculated the Nei measure of pairwise genetic distances using allele frequency data from the 13 STR markers. Polish population data (Pepinski *et al.*, 2005) was included in the analysis as outgroup reference.

The longest distance (0.0320) was noted between the Fujian (a southern) and Henan (a northern) populations, whereas the lowest (0.0041) was observed between Beijing (a northern) and Tianjin (also a northern) populations (Table 1).

**Figure 2:**
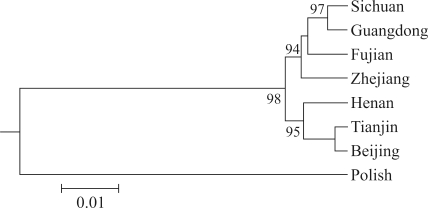
Genetic affinities between seven Chinese populations based on 13 STR loci by DA distance and UPGMA clustering methods.

Based on genetic distance data, population trees were constructed using the UPGMA and Neighbor-Joining methods. As both methods revealed the same pattern, UPGMA results were preferred for display. Bootstrap values for the trees were high (Figure 2). The Sichuan (southern) and the Guangdong (also southern) populations first clustered together with a high bootstrap value (97%), to then cluster with the other two southern populations, the Zhejiang and Fujian, with bootstrap values of 94%. The three northern populations (Beijing, Tianjin and Henan) formed a single cluster with bootstrap values of 95%. The two major populations (the northern and southern) clustered together with bootstrap values of 98%. As expected, on comparing the Polish population, as outgroup control, with any pair of the Chinese populations, the distance was greater.

## Discussion

Owing to the several advantages, such as high polymorphism, ease and low-cost, STR markers have been widely used for fine-scale genetic mapping (Edwards *et al.*, 1991, 1992; Hearne *et al.*, 1992), intra-species phylogenetic reconstruction (Bowcock *et al.*, 1994; Jorde *et al.*, 1998), maternity/paternity determination (Hammond *et al.*, 1994), and forensic analysis (Edwards *et al.*, 1991; Hearne *et al.*, 1992). Consistent with previous studies (Cai *et al.*, 2005; Deng *et al.*, 2007; Ying *et al.*, 2005, 2006), all the 13 STR loci were highly polymorphic in the seven population samples and exhibited desirable values in the forensic analysis and genetic analysis.

Over the past decades, and based on STR polymorphisms, important information has contributed to elucidating the history of human populations (Jorde *et al.*, 1997; Shriver *et al.*, 1997), as well as genetic microdifferentiation among local subdivided populations (Reddy *et al.*, 2001). In the current study, seven Chinese Han populations, with three representative groups from the northern portion and four from the southern, were investigated, by comparing the allele frequency of 13 STR loci, whereby the following consequential information was obtained. First, the 13 loci exhibited high polymorphism in all the seven populations, but with no significant difference in allele distribution in any. It was inferred that both geographical and ethnic affiliations in Chinese Han populations are close. A single STR-based comparison of the population was insufficient to detect the delicate mutual difference among these populations. A method integrating polymorphic information on all the 13 STR loci of each population is essential for determining respective genetic distances. In addition, the specific parameters revealed the high forensic efficiency of the 13 STR loci. Heterozygosity among these ranged from 0.5248 (TPOX in the Henan population) to 0.8989 (D8S1179 in the Zhejiang), whereas the number of alleles observed ranged from 8 (TPOX) to 20 (D18S51). The data presented herein will facilitate calculating matching probabilities in forensic casework, in the event of Chinese individuals being considered as the source of DNA evidence. Furthermore, by using the UPGMA and Neighbor-Joining methods, it was possible to calculate genetic distances on the basis of data from all the 13 STR locus polymorphisms in each population, whereby a population tree was constructed to reflect mutual evolutionary relationships. The results indicated that genetic distances among these populations correspond to their geographic location, Whereas three northern populations formed one cluster, the four southern ones formed another cluster, as confirmed through UPGMA and Neighbor-Joining methodology. Although the distances among the studied populations were only short, clustering remained distinct in certain groups, this being consistent with their ethnohistory and geographic location. Compared to the outgroup control (Polish population), Chinese southern and northern populations clustered together. While clustering tended to occur between two populations with smallest geographic distance, it was notable that the Guangdong population first clustered with that of Sichuan, instead of doing so with the two geographically nearer populations of Fujian and Zhejiang, thereby providing evidence for historical records that the earliest Sichuan population most likely emigrated from Guangdong.

## Supplementary Material

The following online material is available for this article:

Table S1 Genetic polymorphism at the D3S1358 locus for the seven Chinese population groups.

Table S2 Genetic polymorphism at the D16S539 locus for the seven Chinese population groups.

Table S3 Genetic polymorphism at the TPOX locus for the seven Chinese population groups.

Table S4 Genetic polymorphism at the TH01 locus for the seven Chinese population groups.

Table S5 Genetic polymorphism at the CSF1PO locus for the seven Chinese population groups.

Table S6 Genetic polymorphism at the D7S820 locus for the seven Chinesepopulation groups.

Table S7 Genetic polymorphism at the VWA locus for the seven Chinese population groups.

Table S8 Genetic polymorphism at the FGA locus for the seven Chinese population groups.

Table S9 Genetic polymorphism at the D8S1179 locus for the seven Chinese population groups.

Table S10 Genetic polymorphism at the D21S11 locus for the seven Chinese population groups.

Table S11 Genetic polymorphism at the D18S51 locus for the seven Chinese population groups.

Table S12 Genetic polymorphism at the D5S818 locus for the seven Chinese population groups.

Table S13 Genetic polymorphism at the D13S317 locus for the seven Chinese population groups.

This material is available as part of the online article from http://www.scielo.br/gmb.

## Figures and Tables

**Table 1:** Genetic distances of 8 populations using UPGMA software.

Population	Sichuan	Fujian	Guangdong	Tianjin	Zhejiang	Beijing	Henan	Polish
Sichuan								
Fujian	0.0132							
Guangdong	0.0071	0.0146						
Tianjin	0.0188	0.0213	0.0119					
Zhejiang	0.0142	0.0195	0.0149	0.0230				
Beijing	0.0166	0.0193	0.0121	0.0041	0.0210			
Henan	0.0285	0.0320	0.0241	0.0153	0.0318	0.0155		
Polish	0.1202	0.1255	0.1141	0.1099	0.1254	0.1066	0.0980	
