# Supplementary material for: Short-tandem repeat analysis in seven Chinese regional populations
Source: Genet Mol Biol. 2010 Dec 1;33(4):605–9. doi: 10.1590/s1415-47572010000400002 (PMC3036133; doi:10.1590/s1415-47572010000400002)
Supplement: Table S1 — Genetic polymorphism at the D3S1358 locus for the seven Chinese population groups. [file gmb-33-4-605-suppl1.pdf]

Table S1-Genetic polymorphism at the D3S1358 locus for the seven Chinese population groups.

Allele		Southern population				Northern population		
		Sichuan n=260	Fujian n=150	Guangdong n=522	Zhejiang n=147	Tianjin n=150	Beijing n=216	Henan n=101
8							0.0023	
9			0.0033				0.0023	
10			0.0033					
11			0.0067	0.0019				
12				0.0038				
13		0.0019	0.0033	0.0029	0.0034	0.0067	0.0023	
14		0.0577	0.0633	0.0326	0.0238	0.0267	0.0394	0.1089
15		0.3385	0.3067	0.3563	0.2347	0.3733	0.3519	0.3515
16		0.2827	0.3467	0.3008	0.3163	0.2967	0.3333	0.2921
17		0.2635	0.1967	0.2270	0.2619	0.2200	0.2037	0.1337
18		0.0558	0.0567	0.0661	0.1361	0.0733	0.0625	0.0693
19			0.0133	0.0077	0.0170			0.0396
20				0.0010	0.0068	0.0033	0.0023	0.0050
MP		0.1179	0.1200	0.1247	0.0997	0.1365	0.1337	0.0993
PD		0.8821	0.8800	0.8753	0.9003	0.8635	0.8663	0.9007
PIC		0.6809	0.6966	0.6772	0.7158	0.6681	0.6681	0.7180
PE		0.3879	0.5041	0.5081	0.4727	0.4492	0.4786	0.3464
Ho		0.6731	0.7467	0.7490	0.7279	0.7133	0.7315	0.6436
HWE								
df=1	χ^2	4.4493	0.0164	1.3674	0.7998	0.0379	0.1465	7.3252
	P	0.0349	0.8982	0.2423	0.3711	0.8456	0.7019	0.0068

MP: matching probability; PD: power of discrimination; PIC: polymorphism information content
PE: power of exclusion; Ho: heterozygosity; HWE: Hardy-Weinberg equilibrium
