# Supplementary material for: Short-tandem repeat analysis in seven Chinese regional populations
Source: Genet Mol Biol. 2010 Dec 1;33(4):605–9. doi: 10.1590/s1415-47572010000400002 (PMC3036133; doi:10.1590/s1415-47572010000400002)
Supplement: Table S2 — Genetic polymorphism at the D16S539 locus for the seven Chinese population groups. [file gmb-33-4-605-suppl2.pdf]

Table S2-Genetic polymorphism at the D16S539 locus for the seven Chinese population groups.

	Southern population				Northern population		
Allele	Sichuan n=260	Fujian n=150	Guangdong n=522	Zhejiang n=147	Tianjin n=150	Beijing n=216	Henan n=101
6			0.0019		□	□	
8	0.0038	0.0067	0.0134	0.0034	0.0100	0.0116	0.0099
9	0.2885	0.2433	0.2615	0.2959	0.2933	0.2662	0.2574
10	0.1077	0.1167	0.1293	0.1190	0.1067	0.1088	0.1089
11	0.2885	0.3133	0.2490	0.2585	0.2867	0.2662	0.2327
12	0.2077	0.2100	0.2261	0.2211	0.2167	0.2153	0.2376
13	0.0827	0.1000	0.1044	0.0816	0.0700	0.1019	0.1535
14	0.0173	0.0067	0.0125	0.0204	0.0167	0.0231	□
15	0.0019	0.0033	0.0019	□	□	0.0023	□
17	0.0019	□	□	□	□	0.0023	□
22	□	□	□		□	0.0023	□
MP	0.0906	0.0875	0.0739	0.0905	0.0954	0.0786	0.0889
PD	0.9094	0.9125	0.9261	0.9095	0.9046	0.9214	0.9111
PIC	0.7354	0.7395	0.7582	0.7398	0.7310	0.7570	0.7537
PE	0.5098	0.5155	0.5147	0.6041	0.5625	0.5502	0.5486
Ho	0.7500	0.7533	0.7529	0.8027	0.7800	0.7731	0.7723
HWE	□	□	□		□	□	□
df=1 χ^2	0.7961	0.5017	4.6654	0.5157	0.0727	0.4097	0.2271
P	0.3723	0.4788	0.0308	0.4727	0.7875	0.5221	0.6337

MP: matching probability; PD: power of discrimination; PIC: polymorphism information content
PE: power of exclusion; Ho: heterozygosity; HWE: Hardy-Weinberg equilibrium
