# Supplementary material for: Short-tandem repeat analysis in seven Chinese regional populations
Source: Genet Mol Biol. 2010 Dec 1;33(4):605–9. doi: 10.1590/s1415-47572010000400002 (PMC3036133; doi:10.1590/s1415-47572010000400002)
Supplement: Table S3 — Genetic polymorphism at the TPOX locus for the seven Chinese population groups. [file gmb-33-4-605-suppl3.pdf]

Table S3-Genetic polymorphism at the TPOX locus for the seven Chinese population groups.

	Southern population				Northern population		
Allele	Sichuan n=260	Fujian n=150	Guangdong n=522	Zhejiang n=147	Tianjin n=150	Beijing n=216	Henan n=101
6	0.0019						
7			0.0019				
8	0.4904	0.4933	0.5144	0.5433	0.5733	0.4931	0.5396
9	0.1096	0.1167	0.1034	0.1433	0.1133	0.1412	0.1188
10	0.0288	0.0633	0.0287	0.0100	0.0167	0.0185	0.0248
11	0.3327	0.2967	0.3113	0.2900	0.2833	0.3241	0.2673
12	0.0365	0.0300	0.0364	0.0133	0.0133	0.0231	0.0495
13			0.0038				
MP	0.1959	0.1800	0.2017	0.2239	0.2421	0.2046	0.2073
PD	0.8041	0.8200	0.7983	0.7761	0.7579	0.7954	0.7927
PIC	0.5714	0.5948	0.5650	0.5344	0.5140	0.5655	0.5660
PE	0.3244	0.2990	0.3414	0.2386	0.2601	0.3340	0.2101
Ho	0.6269	0.6067	0.6398	0.5533	0.5733	0.6343	0.5248
HWE							
df=1 χ^2	0.0900	1.3749	0.4126	1.4749	0.0245	0.0028	4.1748
P	0.7642	0.2410	0.5207	0.2246	0.8756	0.9577	0.0410

MP: matching probability; PD: power of discrimination; PIC: polymorphism information content
PE: power of exclusion; Ho: heterozygosity; HWE: Hardy-Weinberg equilibrium
