# Supplementary material for: Short-tandem repeat analysis in seven Chinese regional populations
Source: Genet Mol Biol. 2010 Dec 1;33(4):605–9. doi: 10.1590/s1415-47572010000400002 (PMC3036133; doi:10.1590/s1415-47572010000400002)
Supplement: Table S4 — Genetic polymorphism at the TH01 locus for the seven Chinese population groups. [file gmb-33-4-605-suppl4.pdf]

Table S4-Genetic polymorphism at the TH01 locus for the seven Chinese population groups.

Allele	Southern population				Northern population		
	Sichuan n=260	Fujian n=150	Guangdong n=522	Zhejiang n=147	Tianjin n=150	Beijing n=216	Henan n=101
5	0.0019	□	□	□	□	□	□
6	0.0788	0.0867	0.1159	0.0952	0.0933	0.1019	0.1040
7	0.2904	0.2433	0.2730	0.2415	0.2767	0.2523	0.2475
8	0.0404	0.0900	0.0584	0.0680	0.0333	0.0486	0.1139
9	0.5231	0.4967	0.4732	0.5238	0.5533	0.5347	0.4307
9.3	0.0346	0.0267	0.0354	0.0374	0.0133	0.0139	0.0446
10	0.0308	0.0500	0.0441	0.0306	0.0300	0.0440	0.0594
11	□	0.0033	□	0.0034	□	□	□
12	□	0.0033	□	□	□	□	□
13	□	□	□	□	□	0.0046	□
MP	0.2037	0.1518	0.1441	0.1552	0.2064	0.1855	0.1250
PD	0.7963	0.8482	0.8559	0.8448	0.7936	0.8145	0.8750
PIC	0.5787	0.6343	0.6360	0.6084	0.5511	0.5886	0.6862
PE	0.3396	0.3786	0.3897	0.2975	0.2990	0.3786	0.4644
Ho	0.6385	0.6667	0.6743	0.6054	0.6067	0.6667	0.7228
HWE	□	□	□	□	□	□	□
df=1 χ^2	0.0300	0.0809	0.1487	1.4980	0.0019	0.8222	0.0116
P	0.8626	0.7760	0.6998	0.2210	0.9652	0.3645	0.9141

MP: matching probability; PD: power of discrimination; PIC: polymorphism information content
PE: power of exclusion; Ho: heterozygosity; HWE: Hardy-Weinberg equilibrium
