# Supplementary material for: Short-tandem repeat analysis in seven Chinese regional populations
Source: Genet Mol Biol. 2010 Dec 1;33(4):605–9. doi: 10.1590/s1415-47572010000400002 (PMC3036133; doi:10.1590/s1415-47572010000400002)
Supplement: Table S5 — Genetic polymorphism at the CSF1PO locus for the seven Chinese population groups. [file gmb-33-4-605-suppl5.pdf]

Table S5-Genetic polymorphism at the CSF1PO locus for the seven Chinese population groups.

Allele	Southern population					Northern population		
	Sichuan n=260	Fujian n=150	Guangdong n=522	Zhejiang n=147		Tianjin n=150	Beijing n=216	Henan n=101
7	0.0019	0.0033	0.0010	0.0102		□	□	□
8	0.0038	0.0033	0.0019	□		0.0100	0.0046	□
9	0.0596	0.0433	0.0651	0.0612		0.0700	0.0532	0.1089
10	0.2731	0.2833	0.2577	0.2075		0.2533	0.2292	0.1881
11	0.2346	0.2533	0.2347	0.2415		0.2467	0.2500	0.2376
12	0.3404	0.3200	0.3343	0.4082		0.3700	0.3727	0.3564
13	0.0808	0.0933	0.0881	0.0612		0.0400	0.0787	0.0743
14	0.0058	□	0.0134	0.0102		0.0100	0.0069	0.0347
15	□	□	0.0038	□		□	□	□
16	□	□	□	□		□	0.0023	□
17	□	□	□	□		□	0.0023	□
MP	0.1088	0.1100	0.1016	0.1265		0.1111	0.1260	0.0944
PD	0.8912	0.8900	0.8984	0.8735		0.8889	0.8740	0.9056
PIC	0.7011	0.6974	0.7144	0.6814		0.6858	0.6938	0.7269
PE	0.4967	0.4707	0.5414	0.4839		0.3882	0.5502	0.4806
Ho	0.7423	0.7267	0.7682	0.7347		0.6733	0.7731	0.7327
HWE	□		□	□	□	□	□	□
df=1 χ^2	0.0170	0.2659	0.4697	0.0461		2.8100	1.3287	0.6384
P	0.8963	0.6061	0.4931	0.8299		0.0937	0.2490	0.4243

MP: matching probability; PD: power of discrimination; PIC: polymorphism information content
PE: power of exclusion; Ho: heterozygosity; HWE: Hardy-Weinberg equilibrium
