# Supplementary material for: Short-tandem repeat analysis in seven Chinese regional populations
Source: Genet Mol Biol. 2010 Dec 1;33(4):605–9. doi: 10.1590/s1415-47572010000400002 (PMC3036133; doi:10.1590/s1415-47572010000400002)
Supplement: Table S6 — Genetic polymorphism at the D7S820 locus for the seven Chinesepopulation groups. [file gmb-33-4-605-suppl6.pdf]

Table S6-Genetic polymorphism at the D7S820 locus for the seven Chinese population groups.

	Southern population				Northern population		
Allele	Sichuan n=260	Fujian n=150	Guangdong n=522	Zhejiang n=147	Tianjin n=150	Beijing n=216	Henan n=101
6		0.0067					
7	0.0038	0.0033	0.0019			0.0023	
8	0.1942	0.1200	0.1360	0.1259	0.1267	0.1690	0.1089
9	0.0635	0.0900	0.0718	0.0544	0.0633	0.0579	0.0792
9.2				0.0034			
10	0.1365	0.1400	0.1868	0.1599	0.2133	0.1852	0.1881
11	0.3250	0.3000	0.3630	0.4320	0.3233	0.3218	0.2822
12	0.2327	0.3000	0.2117	0.1837	0.2400	0.2176	0.2376
13	0.0327	0.0367	0.0249	0.0340	0.0333	0.0417	0.0693
14	0.0077	0.0033	0.0038	0.0068		0.0023	0.0347
15	0.0038					0.0023	
MP	0.0870	0.0989	0.0915	0.1113	0.0972	0.0863	0.0809
PD	0.9130	0.9011	0.9085	0.8887	0.9028	0.9137	0.9191
PIC	0.7456	0.7434	0.7296	0.7003	0.7354	0.7483	0.7771
PE	0.5919	0.5745	0.5414	0.4839	0.5745	0.6094	0.6978
Ho	0.7962	0.7867	0.7682	0.7347	0.7867	0.8056	0.8515
HWE							
df=1 χ^2	0.3870	0.0501	0.0307	0.0027	0.1428	0.6474	1.2113
P	0.5339	0.8229	0.8609	0.9582	0.7055	0.4210	0.2711

MP: matching probability; PD: power of discrimination; PIC: polymorphism information content
PE: power of exclusion; Ho: heterozygosity; HWE: Hardy-Weinberg equilibrium
