# Supplementary material for: Short-tandem repeat analysis in seven Chinese regional populations
Source: Genet Mol Biol. 2010 Dec 1;33(4):605–9. doi: 10.1590/s1415-47572010000400002 (PMC3036133; doi:10.1590/s1415-47572010000400002)
Supplement: Table S7 — Genetic polymorphism at the VWA locus for the seven Chinese population groups. [file gmb-33-4-605-suppl7.pdf]

Table S7-Genetic polymorphism at the VWA locus for the seven Chinese population groups.

	Southern population				Northern population		
Allele	Sichuan n=260	Fujian n=150	Guangdong n=522	Zhejiang n=147	Tianjin n=150	Beijing n=216	Henan n=101
8	□	□	□	□	□	0.0023	□
9	□	□	□	□	□	0.0023	□
11	□	0.0033	□	□	□	□	□
12	□	□	□	□	□	□	0.0050
13	0.0038	0.0133	□	□	0.0033	0.0046	0.0050
14	0.2635	0.2567	0.2720	0.2789	0.2900	0.2546	0.2475
15	0.0173	0.0367	0.0364	0.0204	0.0400	0.0347	0.0248
16	0.1500	0.1233	0.1916	0.1633	0.1733	0.1667	0.1733
17	0.2135	0.2600	0.2193	0.2619	0.2033	0.2106	0.2228
18	0.2250	0.1833	0.1830	0.1871	0.1900	0.2153	0.2426
19	0.1077	0.0933	0.0872	0.0748	0.0833	0.0926	0.0545
20	0.0173	0.0300	0.0086	0.0102	0.0167	0.0162	0.0248
21	0.0019	□	0.0019	0.0034	□	□	□
MP	0.0704	0.0777	0.0751	0.0808	0.0852	0.0686	0.0726
PD	0.9296	0.9223	0.9249	0.9192	0.9148	0.9314	0.9274
PIC	0.7697	0.7795	0.7685	0.7527	0.7704	0.7784	0.7650
PE	0.5778	0.6883	0.6623	0.5790	0.7413	0.6094	0.5140
Ho	0.7885	0.8467	0.8333	0.7891	0.8733	0.8056	0.7525
HWE	□	□	□	□	□	□	□
df=1 χ^2	0.2659	1.3602	3.7316	0.0003	4.7773	0.0118	1.4232
P	0.6061	0.2435	0.0534	0.9857	0.0288	0.9134	0.2329

MP: matching probability; PD: power of discrimination; PIC: polymorphism information content
PE: power of exclusion; Ho: heterozygosity; HWE: Hardy-Weinberg equilibrium
