# Supplementary material for: Short-tandem repeat analysis in seven Chinese regional populations
Source: Genet Mol Biol. 2010 Dec 1;33(4):605–9. doi: 10.1590/s1415-47572010000400002 (PMC3036133; doi:10.1590/s1415-47572010000400002)
Supplement: Table S8 — Genetic polymorphism at the FGA locus for the seven Chinese population groups. [file gmb-33-4-605-suppl8.pdf]

Table S8-Genetic polymorphism at the FGA locus for the seven Chinese population groups.

	Southern population				Northern population		
Allele	Sichuan<br>n=260	Fujian<br>n=150	Guangdong<br>n=522	Zhejiang<br>n=147	Tianjin<br>n=150	Beijing<br>n=216	Henan<br>n=101
14	□	□	□	□	□	0.0023	□
16	□	0.0100	□	□	□	0.0023	0.0050
17	□	□	□	□	□	0.0023	□
18	0.0327	0.0333	0.0335	0.0408	0.0100	0.0231	0.0050
18.2	0.0019	□	□	□	□	□	□
19	0.0577	0.0467	0.0441	0.0544	0.0667	0.0394	0.0099
20	0.0712	0.0300	0.0536	0.0680	0.0400	0.0509	0.0693
21	0.1231	0.1033	0.1236	0.1190	0.1067	0.0949	0.1139
21.2	□	0.0067	0.0010	0.0034	0.0033	0.0046	0.0149
22	0.1904	0.1467	0.1964	0.1633	0.1967	0.1852	0.1980
22.2	□	0.0100	0.0057	0.0034	0.0133	0.0139	0.0248
23	0.2173	0.2533	0.1830	0.2585	0.1733	0.1875	0.2772
23.2	0.0019	0.0067	0.0077	0.0068	0.0033	0.0069	0.0297
24	0.1558	0.1700	0.1762	0.1361	0.2133	0.2199	0.1436
24.2	0.0019	0.0300	0.0134	0.0034	0.0033	0.0116	0.0198
25	0.0904	0.0800	0.1054	0.0680	0.1233	0.0926	0.0644
25.2	0.0019	0.0067	0.0019	0.0034	□	□	□
26	0.0538	0.0600	0.0489	0.0510	0.0300	0.0417	0.0149
26.2	□	0.0033	□	□	□	□	□
27	□	0.0033	0.0048	0.0136	0.0133	0.0139	0.0050
27.2	□	□	□	0.0034	□	□	□
28	□	□	□	0.0034	□	0.0023	0.0050
29	□	□	0.0010	□	□	□	□
30	□	□	□	□	0.0033	0.0023	□
MP	0.0392	0.0404	0.0372	0.0356	0.0429	0.0393	0.0544
PD	0.9608	0.9596	0.9628	0.9644	0.9571	0.9607	0.9456
PIC	0.8405	0.8451	0.8468	0.8424	0.8346	0.8422	0.8210
PE	0.5990	0.7413	0.7573	0.6822	0.6883	0.7077	0.6783
Ho	0.8000	0.8733	0.8812	0.8435	0.8467	0.8565	0.8416
HWE							
df=1 χ^2	7.2445	0.1575	1.5068	0.3299	0.0756	0.0170	0.0017
P	0.0071	0.6915	0.2196	0.5657	0.7833	0.8962	0.9674

MP: matching probability; PD: power of discrimination; PIC: polymorphism information content
PE: power of exclusion; Ho: heterozygosity; HWE: Hardy-Weinberg equilibrium
