# Supplementary material for: Short-tandem repeat analysis in seven Chinese regional populations
Source: Genet Mol Biol. 2010 Dec 1;33(4):605–9. doi: 10.1590/s1415-47572010000400002 (PMC3036133; doi:10.1590/s1415-47572010000400002)
Supplement: Table S9 — Genetic polymorphism at the D8S1179 locus for the seven Chinese population groups. [file gmb-33-4-605-suppl9.pdf]

Table S9-Genetic polymorphism at the D8S1179 locus for the seven Chinese population groups.

Allele	Southern population				Northern population		
	Sichuan<br>n=260	Fujian<br>n=150	Guangdong<br>n=522	Zhejiang<br>n=147	Tianjin<br>n=150	Beijing<br>n=216	Henan<br>n=101
8	0.0019	□	□	□	0.0033	0.0023	
9	□	□	□	0.0068	□	□	□
10	0.1423	0.1333	0.1303	0.1054	0.1133	0.0926	0.0545
11	0.1154	0.0867	0.1207	0.1020	0.1033	0.0903	0.1386
12	0.1154	0.1600	0.1111	0.1088	0.1167	0.1319	0.1535
13	0.1827	0.1800	0.1762	0.2007	0.1967	0.1875	0.2574
14	0.1942	0.1200	0.1619	0.1871	0.1733	0.1898	0.1782
15	0.1635	0.2000	0.2021	0.1803	0.1767	0.1968	0.1337
16	0.0731	0.1000	0.0814	0.0884	0.0967	0.0972	0.0446
17	0.0115	0.0133	0.0134	0.0204	0.0167	0.0069	0.0396
18	□	0.0067	0.0029	□	0.0033	0.0023	□
19	□	□	□	□	□	0.0023	□
MP	0.0420	0.0432	0.0426	0.0490	0.0444	0.0460	0.0544
PD	0.9580	0.9568	0.9574	0.9510	0.9556	0.9540	0.9456
PIC	0.8313	0.8342	0.8331	0.8326	0.8357	0.8274	0.8142
PE	0.6206	0.6623	0.6475	0.7913	0.7280	0.6623	0.6591
Ho	0.8115	0.8333	0.8257	0.8980	0.8667	0.8333	0.8317
HWE	□	□	□	□	□	□	□
df=1 χ^2	3.2763	0.5646	2.8707	2.3261	0.1349	0.3820	0.0397
P	0.0703	0.4524	0.0902	0.1272	0.7134	0.5365	0.8420

MP: matching probability; PD: power of discrimination; PIC: polymorphism information content
PE: power of exclusion; Ho: heterozygosity; HWE: Hardy-Weinberg equilibrium
