# Supplementary material for: Short-tandem repeat analysis in seven Chinese regional populations
Source: Genet Mol Biol. 2010 Dec 1;33(4):605–9. doi: 10.1590/s1415-47572010000400002 (PMC3036133; doi:10.1590/s1415-47572010000400002)
Supplement: Table S10 — Genetic polymorphism at the D21S11 locus for the seven Chinese population groups. [file gmb-33-4-605-suppl10.pdf]

Table S10-Genetic polymorphism at the D21S11 locus for the seven Chinese population groups.

	Southern population				Northern population		
Allele	Sichuan<br>n=260	Fujian<br>n=150	Guangdong<br>n=522	Zhejiang<br>n=147	Tianjin<br>n=150	Beijing<br>n=216	Henan<br>n=101
26	0.0019	□	□	□	□	□	□
27	0.0019	□	□	□	□	□	□
27.2	□	0.0033	□	□	□	□	□
28	0.0481	0.0300	0.0565	0.0510	0.0400	0.0394	0.0644
28.2	0.0077	0.0100	□	0.0034	0.0067	0.0093	0.0050
29	0.2538	0.2567	0.2548	0.2823	0.2933	0.2894	0.3119
29.2	0.0019	□	□	□	0.0033	□	□
30	0.2673	0.3133	0.2443	0.2857	0.2300	0.2569	0.2574
30.2	0.0077	0.0067	0.0096	0.0204	0.0100	0.0139	0.0149
31	0.0942	0.1100	0.1303	0.0714	0.1167	0.0972	0.1089
31.2	0.0769	0.0367	0.0441	0.0646	0.1000	0.0764	0.0842
32	0.0346	0.0300	0.0546	0.0476	0.0300	0.0324	0.0248
32.2	0.1558	0.1500	0.1370	0.1293	0.1200	0.1366	0.0693
33	0.0019	0.0100	0.0048	0.0034	0.0100	0.0046	□
33.2	0.0404	0.0400	0.0603	0.0306	0.0333	0.0370	0.0594
34	□	□	□	□	□	0.0023	□
34.2	0.0019	0.0033	0.0019	0.0068	0.0067	0.0046	□
35.2	□	□	0.0019	0.0034	□	□	□
36	0.0019	□	□	□	□	□	□
MP	0.0552	0.0897	0.0538	0.0703	0.0563	0.0694	0.0777
PD	0.9448	0.9103	0.9462	0.9297	0.9437	0.9306	0.9223
PIC	0.7972	0.7695	0.8066	0.7822	0.7971	0.7885	0.7799
PE	0.6134	0.6623	0.7036	0.6822	0.5625	0.7354	0.4644
Ho	0.8077	0.8333	0.8544	0.8435	0.7800	0.8704	0.7228
HWE	□	□	□	□	□	□	□
df=1 χ^2	0.3302	1.1012	2.4467	1.1269	1.7965	4.5473	4.7533
P	0.5656	0.2940	0.1178	0.2884	0.1801	0.0330	0.0292

MP: matching probability; PD: power of discrimination; PIC: polymorphism information content
PE: power of exclusion; Ho: heterozygosity; HWE: Hardy-Weinberg equilibrium
