# Supplementary material for: Short-tandem repeat analysis in seven Chinese regional populations
Source: Genet Mol Biol. 2010 Dec 1;33(4):605–9. doi: 10.1590/s1415-47572010000400002 (PMC3036133; doi:10.1590/s1415-47572010000400002)
Supplement: Table S11 — Genetic polymorphism at the D18S51 locus for the seven Chinese population groups. [file gmb-33-4-605-suppl11.pdf]

Table S11-Genetic polymorphism at the D18S51 locus for the seven Chinese population groups.

	Southern population				Northern population		
Allele	Sichuan	Fujian	Guangdong	Zhejiang	Tianjin	Beijing	Henan
	n=260	n=150	n=522	n=147	n=150	n=216	n=101
9	□	0.0033	□	□	□	□	□
10	□	0.0033	□	□	0.0033	0.0023	0.0099
10.2	□	□	□	0.0034	□	□	□
11	0.0019	0.0100	0.0038	0.0068	□	0.0070	0.0099
12	0.0404	0.0233	0.0287	0.0442	0.0267	0.0395	0.0149
13	0.1577	0.1933	0.1983	0.1905	0.2067	0.1837	0.1832
14	0.2058	0.1967	0.1887	0.2109	0.2133	0.2302	0.1634
15	0.1904	0.1400	0.1916	0.1905	0.1667	0.1674	0.1832
16	0.1462	0.1700	0.1264	0.1054	0.1167	0.1372	0.0743
17	0.0712	0.0967	0.0671	0.0646	0.0833	0.0767	0.0792
18	0.0462	0.0233	0.0556	0.0340	0.0500	0.0512	0.0842
19	0.0481	0.0533	0.0575	0.0612	0.0367	0.0302	0.0545
20	0.0308	0.0233	0.0287	0.0408	0.0400	0.0326	0.0396
21	0.0212	0.0200	0.0287	0.0272	0.0300	0.0233	0.0248
22	0.0173	0.0400	0.0144	0.0102	0.0167	0.0070	0.0347
23	0.0135	0.0033	0.0077	0.0068	0.0067	0.0070	0.0149
23.2	□	□	□	□	□	□	0.0248
24	0.0077	□	0.0029	0.0034	□	□	□
25	0.0019	□	□	□	□	0.0023	0.0050
26	□	□	□	□	0.0033	0.0023	□
MP	0.0347	0.0413	0.0388	0.0403	0.0444	0.0427	0.0350
PD	0.9653	0.9587	0.9612	0.9597	0.9556	0.9573	0.9650
PIC	0.8473	0.8440	0.8432	0.8428	0.8402	0.8373	0.8683
PE	0.6798	0.6367	0.7341	0.7498	0.7280	0.7435	0.6027
Ho	0.8423	0.8200	0.8697	0.8776	0.8667	0.8744	0.8020
HWE	□	□	□	□	□	□	□
df=1 χ^2	1.0177	2.2680	0.4468	0.3321	0.0745	0.6151	6.6459
P	0.3131	0.1321	0.5039	0.5644	0.7849	0.4329	0.0099

MP: matching probability; PD: power of discrimination; PIC: polymorphism information content
PE: power of exclusion; Ho: heterozygosity; HWE: Hardy-Weinberg equilibrium
