# Supplementary material for: Short-tandem repeat analysis in seven Chinese regional populations
Source: Genet Mol Biol. 2010 Dec 1;33(4):605–9. doi: 10.1590/s1415-47572010000400002 (PMC3036133; doi:10.1590/s1415-47572010000400002)
Supplement: Table S12 — Genetic polymorphism at the D5S818 locus for the seven Chinese population groups. [file gmb-33-4-605-suppl12.pdf]

**Table S12-**Genetic polymorphism at the D5S818 locus for the seven Chinese population groups.

| | Southern population | | | | Northern population | | |
|---|---|---|---|---|---|---|---|
| Allele | Sichuan<br>n=260 | Fujian<br>n=150 | Guangdong<br>n=522 | Zhejiang<br>n=147 | Tianjin<br>n=150 | Beijing<br>n=216 | Henan<br>n=101 |
| 7 | 0.0308 | 0.0200 | 0.0297 | 0.0408 | 0.0367 | 0.0162 | 0.0198 |
| 8 | 0.0019 | □ | 0.0048 | 0.0034 | □ | 0.0046 | 0.0297 |
| 9 | 0.0846 | 0.0967 | 0.0996 | 0.0646 | 0.0900 | 0.0718 | 0.0545 |
| 10 | 0.1788 | 0.1667 | 0.2165 | 0.1565 | 0.1700 | 0.1944 | 0.1733 |
| 11 | 0.3269 | 0.2933 | 0.3123 | 0.2993 | 0.3300 | 0.2940 | 0.3416 |
| 12 | 0.2327 | 0.2867 | 0.2107 | 0.2959 | 0.2400 | 0.2546 | 0.2228 |
| 13 | 0.1269 | 0.1133 | 0.1044 | 0.1361 | 0.1200 | 0.1458 | 0.1040 |
| 14 | 0.0115 | 0.0133 | 0.0192 | □ | 0.0100 | 0.0185 | 0.0545 |
| 15 | 0.0058 | 0.0100 | 0.0029 | 0.0034 | 0.0033 | □ | □ |
| MP | 0.0805 | 0.0804 | 0.0772 | 0.0964 | 0.0778 | 0.0897 | 0.0913 |
| PD | 0.9195 | 0.9196 | 0.9228 | 0.9036 | 0.9222 | 0.9103 | 0.9087 |
| PIC | 0.7510 | 0.7484 | 0.7585 | 0.7396 | 0.7488 | 0.7508 | 0.7568 |
| PE | 0.4774 | 0.4599 | 0.5828 | 0.5544 | 0.5270 | 0.5837 | 0.3600 |
| Ho | 0.7308 | 0.7200 | 0.7912 | 0.7755 | 0.7600 | 0.7917 | 0.6535 |
| HWE | □ | □ | □ | □ | □ | □ | □ |
| df=1 $\chi^2$ | 4.3703 | 3.5963 | 0.0054 | 0.0011 | 0.4778 | 0.0456 | 11.2602 |
| $P$ | 0.0366 | 0.0579 | 0.9415 | 0.9741 | 0.4894 | 0.8309 | 0.0008 |

MP: matching probability; PD: power of discrimination; PIC: polymorphism information content
PE: power of exclusion; Ho: heterozygosity; HWE: Hardy-Weinberg equilibrium
