# Supplementary material for: Short-tandem repeat analysis in seven Chinese regional populations
Source: Genet Mol Biol. 2010 Dec 1;33(4):605–9. doi: 10.1590/s1415-47572010000400002 (PMC3036133; doi:10.1590/s1415-47572010000400002)
Supplement: Table S13 — Genetic polymorphism at the D13S317 locus for the seven Chinese population groups. [file gmb-33-4-605-suppl13.pdf]

Table S13-Genetic polymorphism at the D13S317 locus for the seven Chinese population groups.

Allele	Southern population				Northern population		
	Sichuan n=260	Fujian n=150	Guangdong n=522	Zhejiang n=147	Tianjin n=150	Beijing n=216	Henan n=101
7	0.0019	□	□	□	□	□	□
8	0.3135	0.3167	0.3065	0.3265	0.2833	0.2546	0.1931
9	0.1365	0.1367	0.1446	0.1463	0.1033	0.1690	0.1188
10	0.1154	0.1400	0.1025	0.1395	0.1733	0.1505	0.1238
11	0.2212	0.2300	0.2423	0.2075	0.2200	0.2199	0.2277
12	0.1692	0.1167	0.1619	0.1395	0.1833	0.1690	0.2228
13	0.0269	0.0367	0.0364	0.0374	0.0300	0.0278	0.0792
14	0.0154	0.0100	0.0057	0.0034	0.0067	0.0093	0.0347
15	□	0.0067	□	□	□	□	□
16	□	0.0033	□	□	□	□	□
18	□	0.0033	□	□	□	□	□
MP	0.0727	0.0797	0.0782	0.0843	0.0769	0.0684	0.0603
PD	0.9273	0.9203	0.9218	0.9157	0.9231	0.9316	0.9397
PIC	0.7611	0.7645	0.7570	0.7584	0.7654	0.7773	0.8005
PE	0.5569	0.6367	0.5828	0.5666	0.5990	0.6445	0.5844
Ho	0.7769	0.8200	0.7912	0.7823	0.8000	0.8241	0.7921
HWE	□	□	□	□	□	□	□
df=1 χ^2	0.3988	0.5289	0.0138	0.0722	0.0016	0.3568	0.9391
P	0.5277	0.4671	0.9066	0.7881	0.9685	0.5503	0.3325

MP: matching probability; PD: power of discrimination; PIC: polymorphism information content
PE: power of exclusion; Ho: heterozygosity; HWE: Hardy-Weinberg equilibrium
